# Exploring Lead-Like Molecules of Traditional Chinese Medicine for Treatment Quest against *Aliarcobacter butzleri*: *In Silico* Toxicity Assessment, Dynamics Simulation, and Pharmacokinetic Profiling

**DOI:** 10.1155/2024/9377016

**Published:** 2024-02-22

**Authors:** Zarrin Basharat, Ibrar Ahmed, Sulaiman Mohammed Alnasser, Alotaibi Meshal, Yasir Waheed

**Affiliations:** ^1^Alpha Genomics (Private) Limited, Islamabad 45710, Pakistan; ^2^Group of Biometrology, The Korea Research Institute of Standards and Science (KRISS), Yuseong District, Daejeon 34113, Republic of Korea; ^3^Department of Pharmacology and Toxicology, Unaizah College of Pharmacy, Qassim University, Buraydah 52571, Saudi Arabia; ^4^Department of Pharmacy Practice, College of Pharmacy, University of Hafr Al Batin, Hafar Al Batin, Saudi Arabia; ^5^Office of Research, Innovation and Commercialization (ORIC), Shaheed Zulfiqar Ali Bhutto Medical University (SZABMU), Islamabad 44000, Pakistan; ^6^Gilbert and Rose-Marie Chagoury School of Medicine, Lebanese American University, Byblos 1401, Lebanon

## Abstract

**Background:**

*Aliarcobacter butzleri* is a Gram-negative, curved or spiral-shaped, microaerophilic bacterium and causes human infections, specifically diarrhea, fever, and sepsis. The research objective of this study was to employ computer-aided drug design techniques to identify potential natural product inhibitors of a vital enzyme in this bacterium. The pyrimidine biosynthesis pathway in its core genome fraction is crucial for its survival and presents a potential target for novel therapeutics. Hence, novel small molecule inhibitors were identified (from traditional Chinese medicinal (TCM) compound library) against it, which may be used for possible curbing of infection by *A. butzleri. Methods*. A comprehensive subtractive genomics approach was utilized to identify a key enzyme (orotidine-5′-phosphate decarboxylase) cluster conserved in the core genome fraction of *A. butzleri*. It was selected for inhibitor screening due to its vital role in pyrimidine biosynthesis. TCM library (*n* > 36,000 compounds) was screened against it using pharmacophore model based on orotidylic acid (control), and the obtained lead-like molecules were subjected to structural docking using AutoDock Vina. The top-scoring compounds, ZINC70454134, ZINC85632684, and ZINC85632721, underwent further scrutiny via a combination of physiological-based pharmacokinetics, toxicity assessment, and atomic-scale dynamics simulations (100 ns).

**Results:**

Among the screened compounds, ZINC70454134 displayed the most favorable characteristics in terms of binding, stability, absorption, and safety parameters. Overall, traditional Chinese medicine (TCM) compounds exhibited high bioavailability, but in diseased states (cirrhosis, renal impairment, and steatosis), there was a significant decrease in absorption, Cmax, and AUC of the compounds compared to the healthy state. Furthermore, MD simulation demonstrated that the ODCase-ZINC70454134 complex had a superior overall binding affinity, supported by PCA proportion of variance and eigenvalue rank analysis. These favorable characteristics underscore its potential as a promising drug candidate.

**Conclusion:**

The computer-aided drug design approach employed for this study helped expedite the discovery of antibacterial compounds against *A. butzleri*, offering a cost-effective and efficient approach to address infection by it. It is recommended that ZINC70454134 should be considered for further experimental analysis due to its indication as a potential therapeutic agent for combating *A. butzleri* infections. This study provides valuable insights into the molecular basis of biophysical inhibition of *A. butzleri* through TCM compounds.

## 1. Introduction

*Aliarcobacter butzleri* was first described in 1991 and is found in water, soil, and various food sources [1]. It is considered an emerging foodborne pathogen and has been associated with a wide range of gastrointestinal diseases [2]. The pathogenesis of *A. butzleri* is not fully understood, but it is thought to involve a range of virulence factors, including adhesins, invasins, and toxins [3]. The organism is known to colonize the intestinal tract and has been shown to adhere to and invade intestinal epithelial cells [4]. It produces a variety of toxins, including cytotoxins, enterotoxins, and hemolysins, which can cause damage to host cells and tissues [5]. It can survive in harsh environments, such as low pH, high salt concentrations, and low oxygen levels, which makes it difficult to control and prevent infection [6]. The organism is known to be resistant to a variety of antibiotics, including macrolides, fluoroquinolones, and tetracyclines [7]. The diagnosis of *A. butzleri* infections is usually based on the isolation of the bacteria from clinical samples and the identification by laboratory methods such as microscopy, culture, and molecular biology [8]. Diagnosis of *A. butzleri* infection can be challenging, as the organism is not easily detectable using standard microbiological methods. However, molecular methods, such as PCR and DNA sequencing, can be used to identify the organism in clinical and environmental samples [9].

Whole genome of *A. butzleri* has been sequenced from various sources, like cattle [10], milk [11], poultry [12], shellfish [13], and diseased humans [14]. Pan-genome on 32 strains of this bacterium has previously been attempted and shown to have a hypervariable accessory genome [15]. Additionally, virulence and immune response inciting genes have been identified. Pan-genomics, which is the study of the entire genetic repertoire of a group of organisms, has previously revealed the existence of conserved gene clusters that are present in multiple strains of a species and are important for drug design [16]. By targeting such gene clusters, a broad-spectrum inhibitor may be obtained that could be effective against multiple strains of a pathogenic bacterial specie, including strains that have developed resistance to existing treatments. Previously, efflux pumps in the core genome have been identified as drug target in Yersinia sp., using pan-genomics, and used for identifying inhibitors from traditional medicinal compounds [17]. In another study, L-lysine biosynthesis pathway protein was targeted using a subtractive core genomics approach and phytochemical inhibitors were identified against Orientia sp. [18].

Phytochemicals, which are naturally occurring compounds found in plants, have been found to have antibacterial properties and may be used as an alternative or alongside antibiotics for synergistic impact in some resistant isolates [19]. Phytochemicals may be better than antibiotics in curbing bacteria due to their broader spectrum of activity and lower risk of resistance development due to their multiple mechanisms of action [20]. Thus, bacteria may be less likely to develop resistance to them compared to antibiotics, which typically target a single bacterial pathway. Some phytochemicals have been found to have synergistic effects when combined with antibiotics, which can enhance the effectiveness of antibiotics. Antibiotics can have side effects such as gastrointestinal disturbances, allergic reactions, and drug interactions [21]. Phytochemicals from traditionally used medicinal plants are generally considered safer with fewer side effects, due to their innocuous usage by humans since long times [22].

Traditional Chinese medicine (TCM) has a long history of use in the treatment of infectious diseases, and many TCM compounds have been shown to have antibacterial activity [23]. In recent years, virtual screening methods have been used to identify TCM compounds that may be effective in inhibiting bacteria [24]. Some examples of TCM compounds that have been identified using virtual screening methods include antibiofilm agents [25], purine synthesis inhibition [26], and amino acid synthesis inhibition [27] in pathogenic bacteria. In pursuit, the present study attempted to identify drug-like compounds from TCM library against ODCase enzyme of *A. butzleri*. Dynamics simulation was conducted to assess stability of these compounds while ADMET profiling was done for toxicity assessment. PBPK modeling was also carried out for determining possible bioavailability and other parameters of importance, required for effectiveness as a drug.

## 2. Material and Methods

The phytochemical inhibitor prioritization approach consisted of several steps outlined below:

### 2.1. Pan-genomics

Pan-genome of *A. butzleri* was accessed from ProPan database (https://ngdc.cncb.ac.cn/propan/pananalysis/28197; accessed 16 Feb 2023). The database integrates information on pan-genomes (the collection of all genes present in a set of genomes from a given species or population) and gene families from over 2000 prokaryotic species [28]. The database allows users to browse the genomes of individual prokaryotic species and visualize the distribution of gene families and other genomic features. It is designed to provide a comprehensive overview of the evolution and diversity of prokaryotic genomes and to facilitate comparative genomics and functional annotation. Nucleotide diversity analysis and COG function categories were obtained for gene clusters for *A. butzleri*. In total, 86.7 MB gene file, with a total 9870 gene clusters from 42 strains (listed in Table 1), was obtained. They were differentiated into core, unique, and pan-fraction gene clusters. Core clusters (*n* = 1272) were subjected to in-house subtractive genomics pipeline in bash, according to parameters described in the previous studies [29, 30]. Initially, redundant sequences were removed by CD-Hit. Remaining sequences were BLAST against DEG and CEG databases for essentiality analysis. Translated product of common genes from these databases was then BLAST against human proteome and, later, DrugBank. Obtained hits were labelled as drug targets.

### 2.2. Target Selection and Inhibitor Screening

Among the core gene clusters identified as drug targets, one depicting ODCase (cluster 1186) from pyrimidine metabolism pathway was selected for further downstream analysis based on its novelty, utility, and importance for the bacterial survival. 3D structure of the protein was modeled using SWISS-model [31] and assessed using ERRAT at SAVES server (https://saves.mbi.ucla.edu/) and Ramachandran plot through PDBSum server (http://www.ebi.ac.uk/thornton-srv/databases/pdbsum/). Ligand and protein structure was prepared before docking by assigning bond orders, protonating, and adding missing atoms and energy minimization [32]. Orotidylic acid, natural binder of ODCase [33], was taken as control for docking. TCM library of 36,000 molecules was filtered for drug-like candidates using Lipinski's druggability filter and 11,431 compounds fulfilling the criteria were subjected to pharmacophore generation, according to the parameters described previously [34]. Screening against the obtained 77 compounds was then carried out using AutoDock Vina. Fpocket was used for pocket calculation, and pocket parameters were 3-6 Å radius with druggability score between 0 and 1 and volume of 100-2000 Å^3^. Box offset of 12 Å and pocket having a druggability score 0.8 and volume 366 were used for analysis. Only the pose with best binding affinity was retained. Interactions were mapped, and PRODIGY (PROtein binDIng enerGY prediction) server (https://bianca.science.uu.nl/prodigy/lig; accessed 9 July 2023) was used for calculating Δ*G* (kcal/mol) of the complexes.

### 2.3. ADMET Profiling

The PKCSM tool (https://biosig.lab.uq.edu.au/pkcsm/; accessed on 18 Feb, 2023) was used to conduct ADMET analysis. This tool utilizes graph-based machine learning and consists of 14 regression models and 16 classification models [35]. These models predict various pharmacokinetic or toxicity properties and categorize outcomes into two classes, respectively. The ADMET descriptors evaluated in this analysis include caco-2 permeability, intestinal absorption in humans, BBB permeability, cytochrome inhibition, renal enzyme substrate, total clearance, Rat LD50, and Ames toxicity [36]. The SMILE string was used as input for the evaluation of these parameters, and the obtained results were compared for top compounds.

### 2.4. Pharmacokinetics

To simulate the physiological-based pharmacokinetic (PBPK) parameters of a 100 mg oral tablet of the compound, a compartmental model was developed using GastroPlus software (version 9.8.2, Simulation Plus, Inc., Lancaster, PA, USA). The simulation was conducted on human subjects in a fasting prandial state, with two sets of population taken into account. The first set consisted of 100 healthy individuals with an age range of 20-60 years, measure time = 10 h, and a pH of 7.2. The second set consisted of 100 cirrhotic, 100 renally impaired, and 100 people having steatosis. Additionally, 100 pregnant women with an age range of 20-50 years were put in this group. Physiological measurements for various parameters were accounted (Supplementary Figure 1), and to ensure consistency with the previous studies [24, 37], the particle radius, dissolution model, and density were kept constant. To determine the pKa values of the compound, the ADMET profiler version 10 was utilized. A fixed first pass was assumed for the liver, and a separate jejunal and paracellular permeability model was included. A persistent electrical potential gradient was assumed for the length of the intestinal tract to enhance accuracy. The simulation included the calculation of the compound's bioavailability, absorption, and plasma concentration to aid in determining optimal dosing. Mean of the values obtained for 100 runs of simulations for bioavailability, time to reach the maximum concentration in plasma, area under curve, etc., were taken and analyzed.

### 2.5. Dynamics Simulation

In order to gain a more comprehensive understanding of the interaction and stability of the top complexes, dynamics simulations were conducted using the Desmond software from Schrodinger LLC. To ensure the geometries were correct, OPLS3e force field was employed and energy minimization was performed. The TIP3P water solvation model was selected in an orthorhombic box for boundary [38]. The complexes were neutralized by adding Na+ ions and used a salt concentration of 0.15 M. The simulation lasted for 100 ns with a recording interval of 10 ps and an energy of 5. The standard NPT ensemble class was used with a pressure of 1.01325 bar and a temperature of 300 K. After the simulation, interaction evaluation was done, to gain more insights. The Bio3D module of the R package [39] was used to conduct post-MD analysis. To align the trajectory to reference frame 0, the fit.xyz() function was employed. The resulting data was then used to generate plots of RMSD and principal component analysis (PCA). PC1 and PC2 were examined for visualizing the conformational dynamics [39].

## 3. Results

### 3.1. Pan-genome Analysis

The pan-genome was open and comprised of 1271 core gene clusters (12.88%), 3556 dispensable (36.03%), and 5,043 unique (51.09%) gene clusters. Maximum nucleotide polymorphism amounted to 0.11 in core and 0.40 in pan-genome (Supplementary Figure 2A). Lowest number of accessory genes were in strain 16CS0821-2 (*n* = 718) and highest in RMI (*n* = 1043). Unique gene count was lowest (*n* = 4) in three strains RM4018, L350, and L352 while highest in Ab_CR1132 (*n* = 424) (Table 1).

Most of the core genes were involved in translation, ribosomal structure, and protein genesis, while most of the dispensable genes were involved in replication, recombination, and repair (Supplementary Figure 2B). Majority of these proteins had an unknown function.

### 3.2. Drug Target Selection

Targeting the core genome of a bacterial species for drug development can be advantageous for drug targeting due to its conservation of function, essentiality, and broader coverage [40]. Core fraction revealed 2123 nonredundant protein sequences. DEG and CEG homologs were 947 and 855, respectively. Common sequences from both these databases (*n* = 821) and dissimilar to human proteome were 398. Among these, 163 were dissimilar to gut proteome, while 59 matched DrugBank sequences, and 49 of these were linked with various KEGG pathways (Supplementary Table 1). Pyrimidine metabolism-related pathway enzymes were only two (WP_014468039.1 and WP_198407793.1). Cluster number 1186 was selected, depicting ODCase (accession no. WP_198407793.1) for further analysis. ODCase is considered a promising target for the development of antimicrobial and antifungal agents as it is involved in the biosynthesis of pyrimidine nucleotides, which are a class of nucleotides that include cytosine, uracil, and thymine [41, 42]. These nucleotides are essential building blocks of DNA and RNA. Inhibition of ODCase can lead to the accumulation of toxic intermediates in the pyrimidine biosynthesis pathway, which can ultimately result in cell death [43].

#### 3.2.1. Structure Modeling and Docking

**Figure 1:**
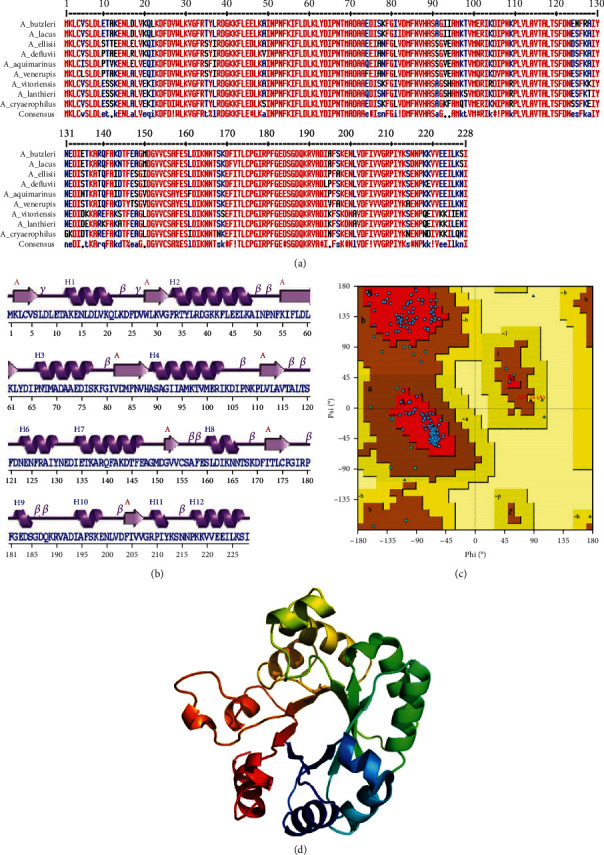
(a) Multiple sequence alignment of ODCase enzyme, showing conservation in closely related Aliarcobacter species. (b) Secondary structure depiction of ODCase. (c) Ramachandran plot showing high quality of modeled structure with >90% residues in core allowed region. (d) 3D modeled structure with one sheet, beta-alpha-beta units, helices, and turns. The C- and N-terminus of the protein is represented by –C in red and –N in blue, respectively.

Modeled structure (Figure 1(a)) consisted of one sheet (with eight strands of topology -1X -1X -1X -1X -1X -1X -1X), seven beta-alpha-beta units, two beta bulges, 12 helices, nine helix-helix interacs, 15 beta turns, and two gamma turns. Overall ERRAT quality factor was 99.07, while Ramachandran plot (Figure 1(b)) showed 91.8% residues in core allowed, 7.7% in allowed, 0.5% in generously allowed, and not even a single residue in disallowed regions.

**Figure 2:**
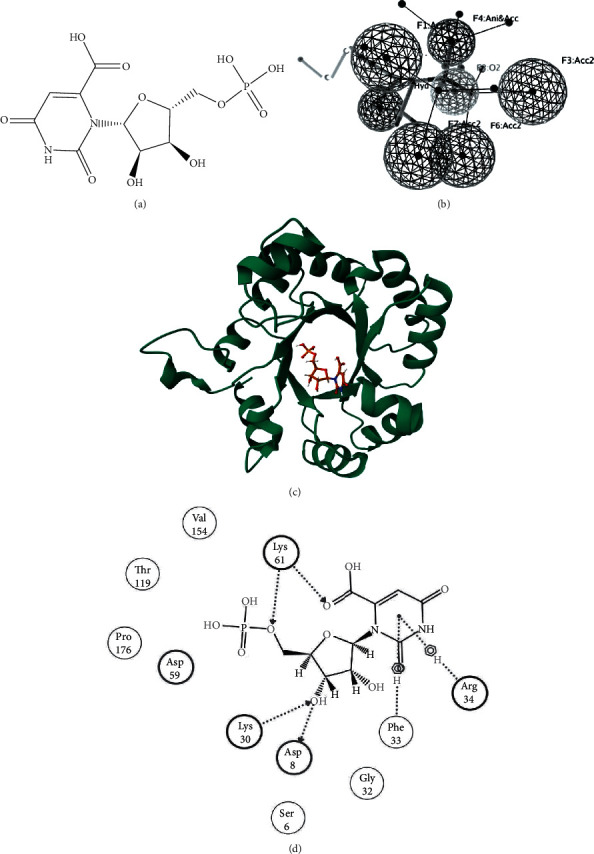
(a) 2D depiction of orotidylic acid. (b) Pharmacophore features of orotidylic acid. (c) 3D depiction of ODCase bound with orotidylic acid. (d) 2D depiction of orotidylic acid and interacting residues of ODCase.

Orotidylic acid was taken as control (Figure 2(a)) and used for generating seven pharmacophoric features (Figure 2(b)).

**Figure 3:**
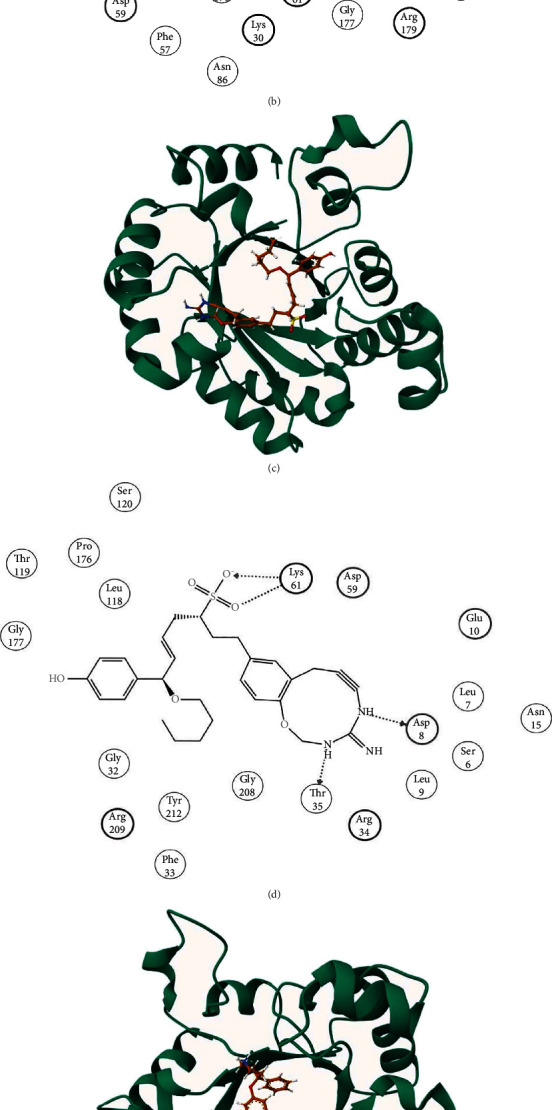
(a) 3D interaction of ZINC70454134 with ODCase. (b) 2D interaction of ZINC70454134 with ODCase. (c) 3D interaction of ZINC85632684 with ODCase. (d) 2D interaction of ZINC85632684 with ODCase. (e) 3D interaction of ZINC85632721 with ODCase. (f) 2D interaction of ZINC85632721 with ODCase.

Docking of the ODCase with orotidyclic acid (Figure 2(c)) gave a score of -3.39 (Table 2) and made interactions with 11 residues of ODCase, including two acidic, three basic, and six hydrogen bond interactions (Figure 2(d)). The binding affinity scores of best binding top three TCM compounds were -4.30 kcal/mol (ZINC70454134; IUPAC name: (3S,8S,9S,10R,13S,14R,17R)-17-[(1R)-2-hydroxy-1-[(2R)-5-(methoxymethyl)-4-methyl-6-oxo-2,3-dihydropyran-2-yl]ethyl]-10,13-dimethyl-1-oxo-2,3,4,7,8,9,11,12,14,15,16,17-dodecahydrocyclopenta[a]phenanthrene-3-sulfonic acid), -4.2 kcal/mol (ZINC85632684; IUPAC name: (E,3R,7R)-1-(5-amino-2-oxa-4,6-diazabicyclo[8.4.0]tetradeca-1(10),5,11,13-tetraen-7-yn-12-yl)-7-(4-hydroxyphenyl)-7-pentoxyhept-5-ene-3-sulfonic acid), and -4.02 kcal/mol (ZINC85632721; IUPAC name: (E,3R,7R)-7-hydroxy-7-(4-hydroxyphenyl)-1-[4-(4-phenylpiperidin-4-yl)oxyphenyl]hept-5-ene-3-sulfonic acid), depicting stronger binding compared to the control. ZINC70454134 and ODCase complex (Figure 3(a)) made one acidic and four basic interactions (Figure 3(b)). Five interactions were conserved between control and ZINC70454134 binding with ODCase (Asp59, Lys61, Val154, Thr119, and Pro176). ZINC85632684 and ODCase complex (Figure 3(c)) made three acidic and three basic interactions (Figure 3(d)). Except for Arg154, all other interactions were conserved between control and ZINC85632684. ZINC85632721 and ODCase complex (Figure 3(e)) made 16 interactions (Figure 3(f)). Except for Ser6, the rest of the interactions were similar between ZINC85632721 and control with ODCase.

### 3.3. ADMET Profiling

Best hits (ZINC70454134, ZINC85632684, and ZINC85632721) were subjected to ADMET profiling, along with control orotidylic acid. Predicted values for various pharmacokinetic and toxicity parameters were obtained and compared (Table 3). Water solubility of prioritized TCM compounds decreased in the order of ZINC70454134, ZINC85632684, and ZINC85632721. The Caco2 permeability across the intestinal lining was highest for ZINC70454134, followed by ZINC85632721 and then ZINC85632684.

VDss represents the theoretical volume into which a drug is distributed in the body, assuming it is uniformly distributed in plasma and tissues at equilibrium [44]. A negative value for VDss suggests that the drug is mainly confined to plasma. Fraction unbound refers to the proportion of a drug that is unbound or free in plasma and available for distribution to tissues [45]. A higher value for fraction unbound suggests that a larger proportion of the drug is available to exert its pharmacological effects. ZINC70454134 was predicted to be more restricted to plasma. It also had higher clearance or elimination from the body compared to ZINC85632684 and ZINC85632721. All the TCM compounds were a substrate of just one class of CYP450 enzymes, i.e., CYP3A4. Ames toxicity was null for all compounds. ZINC70454134 showed maximum tolerated dose in humans and no skin sensitization. Based on the data in Table 3, it appears that ZINC70454134 has the most favorable toxicity profile among the four compounds. It has a higher oral rat chronic toxicity LOAEL value than ZINC85632684 and ZINC85632721 and does not have any hepatotoxicity.

### 3.4. PBPK Analysis

PBPK was simulated under two conditions, normal (including male and female without any other disease), steatosis, cirrhosis, renal impairment, and in pregnant women. ZINC70454134 had the highest *F* (%) and maximum concentration in blood (Cmax) values among the three compounds, indicating a relatively high degree of bioavailability and exposure in both normal and pregnant condition (Table 4). Time to reach peak exposure was almost the same for all compounds in both conditions. All the three TCM compounds had different values for Fa (%) and FDp (%) under normal and pregnant conditions, but the variation was not drastic. Under normal conditions, TCM compounds had a relatively high bioavailability, indicating that a large fraction of the administered dose reached the systemic circulation, but this value was lower in pregnant condition. This indicates a need for dosage adjustments in pregnant females. However, the peak concentration in blood, and the hours to reach peak exposure did not have much difference. The AUC values indicated a high total exposure to the compound over time and were even higher in pregnant condition. These results suggest a relatively high bioavailability and exposure levels under both normal and pregnant conditions, but a higher exposure under pregnant conditions. The first-pass metabolism did not show a large variation, and AUC values were notably lower in diseased patients, indicating reduced total exposure to the drugs over time. Overall, cirrhosis, renal impairment, and steatosis led to a pronounced decrease in absorption, Cmax, and AUC. Understanding these differences is important for tailoring drug regimens to individual patients with these specific medical conditions.

### 3.5. Dynamics Simulation

Simulation of control bound with ODCase as well as top-scoring compounds ZINC70454134, ZINC85632684, and ZINC85632721 was conducted. 28,202 atoms of the control-ODCase, 28,266 atoms of the ODCase-ZINC70454134, 28,238 atoms of the ODCase-ZINC85632684, and 28,271 atoms of the ODCase-ZINC85632721 complex, with respective 8154, 8161, 8153, and 8165 water molecules, were simulated in a box.

**Figure 4:**
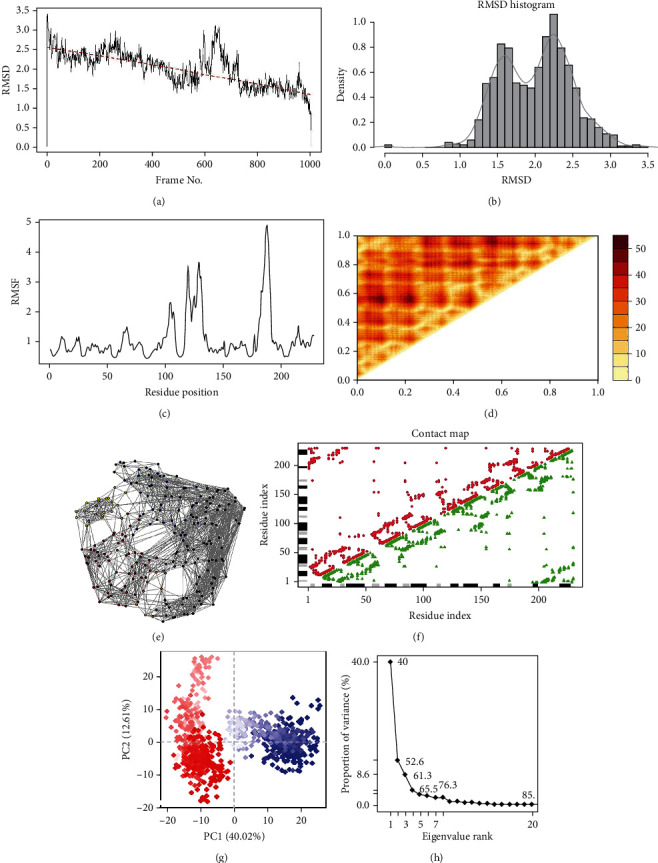
(a) RMSD plot of ODCase with orotidylic acid over 100 ns. (b) Histogram of overall RMSD of the complex. (c) RMSF of ODCase with orotidylic acid. (d) Distance matrix of the complex. (e) Coarse grain network of protein bound with orotidylic acid. (f) Contact map plot of the complex. (g) PC1 and PC2 plot of the complex trajectory for 100 ns. Colour is from beginning (blue) to end (red) of simulation. (h) Proportion of variance plot for various principal coordinates for the trajectory of the complex.

**Figure 5:**
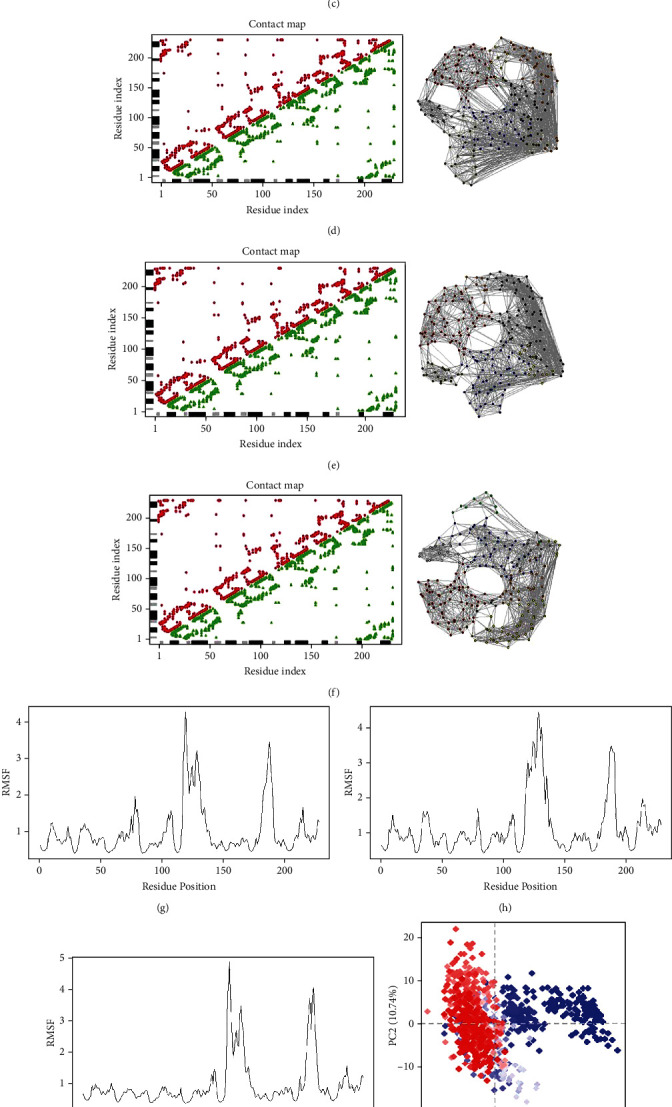
(a) RMSD plot of ODCase with ZINC70454134 over 100 ns. (B) RMSD plot of ODCase with ZINC85632684 over 100 ns. (c) RMSD plot of ODCase with ZINC85632721 over 100 ns. (d) Contact map plot of ODCase with ZINC70454134 alongside coarse grain network map of ligand bound protein. (e) Contact map plot of ODCase with ZINC85632684 alongside coarse grain network map of ligand bound protein. (f) Contact map plot of ODCase with ZINC85632721 alongside coarse grain network map of ligand bound protein. (g) RMSF of ODCase-ZINC70454134 complex. (h) RMSF of ODCase-ZINC85632684 complex. (i) RMSF of ODCase-ZINC85632721 complex. (j) PC1 and PC2 coordinate map of trajectory for ODCase-ZINC70454134 complex. (k) PC1 and PC2 coordinate map of trajectory for ODCase-ZINC85632684 complex. (l) PC1 and PC2 coordinate map of trajectory for ODCase-ZINC85632721 complex. Colour is from beginning (blue) to end (red) of simulation.

On the average, the RMSD did not exceed 3 Å for control-ODCase (Figures 4(a) and 4(b)), while the region around residue 130, 160, and 180 showed fluctuations larger than the rest of the ODCase (Figure 4(c)). These regions comprised of helix and loop region. The RMSD of ZINC70454134 and ZINC85632721 did not exceed 2.5 Å, but the RMSD of the ZINC85632684-ODCase complex reached up to 3 Å during 40-60 ns interval (Figures 5(a)–5(c)). However, it decreased and became stable after 70 ns and kept around 1.5 Å afterwards. Lys30 and Leu60 made hydrogen bonds while Asp59 made a water bridge with the control compound, throughout the simulation time (Supplementary Figure 3A). Apart from this, Lys30, Asp59, Lys61, Pro176, Arg179, and Lys189 retained contact with ligand throughout simulation (Supplementary Figure 3B). No hydrogen bond or other interaction was retained for whole time of the simulation for ZINC70454134 (Supplementary Figure 4A, 4B). Leu7 and Glu10 retained hydrogen bond with ZINC85632684 for entire simulation time, along with interaction with Asp8 for more than 30% of simulation time (Supplementary Figure 4C, 4D). ODCase made interactions like hydrogen bond and water bridges with ZINC85632721 but did not retain for the entire time of simulation (Supplementary Figure 4E). Asp64 made interaction for more than 30% of time (Supplementary Figure 4F).

PCA and relative proportion of variance were calculated for all complexes. PC1 accounted for 31, ~36, and ~33%, while PC2 accounted for approximately 11, 20, and 20% of the total variance for ZINC70454134, ZINC85632684, and ZINC85632721, respectively. Proportion of variance (Supplementary Figure 5) was the amount of variability in the data explained by each principal component. The eigenvalue rank, on the other hand, referred to the relative importance of each principal component in explaining the overall variability of the data. PC1 captured the largest proportion of the variance, indicating that it explains most of the variability in the data. This could be due to large-scale conformational changes in the protein-ligand complex or due to fluctuations in the binding site caused by the ligand. In this scenario, the second principal component captured a smaller proportion of the variance but still was important in explaining the overall variability of the data. A higher eigenvalue rank of PC2 vs. PC1 indicates that it captures a more subtle, but still significant, aspect of the protein-ligand conformational dynamics, and the variation was higher for all complexes. Thus, all complexes had overall best binding affinity, visible from simulation results. The PCA proportion of variance and eigenvalue rank also validate this. Both of these are related but they do not always perfectly align. Nevertheless, they are important in understanding the contribution of each principal component to the overall variability of the protein-ligand complex's conformational dynamics. In summary, the compounds showed stable binding, with average RMSD less than 3 Å over the course of 100 ns. The simulation replicates of the complexes showed the same dimensions of the trajectories and, hence, RMSD (Supplementary Figure 6).

## 4. Discussion

*A. butzleri* is allied with *Campylobacter* spp. and is a zoonotic pathogen [11]. It can cause diarrhea, nondiarrheal gastrointestinal illness, and arcobacteriosis [46]. Treatment of *A. butzleri* infection typically involves the use of antibiotics, such as fluoroquinolones and macrolides, but resistance to these antibiotics is becoming increasingly common [2, 7]. Therefore, prevention of *A. butzleri* infection through improved hygiene and sanitation practices, as well as proper food handling and preparation, is essential to control the spread of this emerging pathogen. Additionally, new compounds that could serve as drug for curbing *A. butzleri* infection are needed. For this purpose, a pan-genomics-allied subtractive genomics approach was used to identify novel therapeutic target and screen TCM natural product inhibitors against it.

Pan-genomics is a field of study that focuses on comparing the complete genetic makeup of individuals from a given population or species [47]. Understanding the pan-genome of a bacterial species can provide insights into its evolution, virulence, and antibiotic resistance potential, which can inform the development of new diagnostic tools, vaccines, and treatments [48]. One key application of pan-genomics in bacterial drug target identification is targeting the core fraction due to conservation and similar structures across different strains [17]. The percentage of core genes for *A. butzleri* was just 12.88%, while the higher percentage of dispensable and unique genes in the pan-genome, i.e., 36.03% and 51.09%, respectively, is likely due to horizontal gene transfer and genetic drift. The core fraction of *A. butzleri* comprised the set of genes present in all strains of this species, most of which were essential for the survival and replication and involved in basic metabolic processes and other fundamental functions. Among these, focus was on pyrimidine biosynthesis pathway enzyme cluster in *A. butzleri*, as it represents a promising target for the development of new drugs [49]. This pathway is essential for bacterial growth due to production of the building blocks for DNA/RNA synthesis and is highly conserved in bacteria, making it an attractive target for developing broad-spectrum inhibitors [50]. ODCase was selected in this pathway for its novelty as it is not much explored against phytochemical based inhibitors. It is a member of the family of pyridoxal 5′-phosphate- (PLP-) dependent enzyme [51] and catalyzes the decarboxylation of orotidine 5′-phosphate (OMP) to uridine 5′-monophosphate (UMP), which is an important precursor for the synthesis of pyrimidine nucleotides [52].

**Figure 6:**
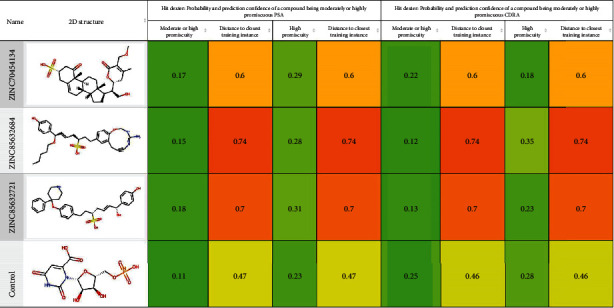
Machine learning-based probability and confidence assessment of the top-scoring ligand promiscuity. Source Hit Dexter 2.0 (https://nerdd.univie.ac.at/; accessed 10 September 2023).

Several classes of ODCase inhibitors have previously been identified, including ginkgolide [53] and novel C6 uridine replacements for antimalarial activity [54]. Anticancer activity of 6-azido-5-fluoro and 5-fluoro-6-iodo derivatives [55] has also been reported. However, to the best of authors' knowledge, TCM-based discovery of phytochemicals against the ODCase of any pathogenic bacteria has not been reported yet. Therefore, the goal of this study was to identify natural compounds from TCM library that may be used as lead compounds for the development of new drugs against ODCase of *A. butzleri*. Pharmacophore mapping and then molecular docking were performed for screening a compound quickly and efficiently. This method helped identify potential inhibitors before their synthesis or isolation. Best docked compounds were ZINC70454134, ZINC85632684, and ZINC85632721, depicting stronger binding compared to the control. Their promiscuity was assessed to see if they could interact with multiple biological targets or proteins. However, they were nonpromiscuous and are best fit for inhibition of ODCase only (Figure 6).

MD simulation can help identify conformational changes in ligand binding site and even cryptic or allosteric binding sites [56]. It also helps predict small molecule binding energies and allow for the introduction of protein flexibility before or after a docking protocol, refining the structure of protein-ligand complexes in various environments, and ranking complexes with more accurate binding energy calculations [57]. The observed fluctuations in conformation and stability over simulation time provide dynamic view of interactions in drug development. A compound that maintains stability and favorable interactions with the target protein in a cellular environment is more likely to exhibit the desired therapeutic effects due to its continued occupation of the protein site. Salt concentration and overall environment can also influence electrostatic behaviour of a drug binding with receptor and simulation mimics that, along with watery environment of a cell which impact dissolution of a drug. Results suggest that ODCase-ZINC70454134 complex has a better overall binding affinity (RMSD < 3 Å) than ODCase-ZINC85632684 complex, which is supported by the PCA proportion of variance and eigenvalue rank as well (Supplementary Figure 5). Hence, the stable binding and favorable conformational changes observed in the simulations suggest that ZINC70454134 has potential as a drug candidate.

The favorable outcomes in the computer-aided drug design study against *A. butzleri*, particularly with a focus on ZINC70454134 as an inhibitor, can be attributed to its optimized binding, stability, absorption, and safety parameters, distinguishing it among the screened compounds. Its superior characteristics, as evidenced by its distinct bioavailability compared to other TCM compounds, highlight its potential as a promising drug candidate. The observed impact of diseases such as cirrhosis, renal impairment, and steatosis on decreased absorption, Cmax, and AUC of compounds also underscores the importance of considering physiological conditions in drug performance evaluation. Moreover, MD simulation results supported the superior binding affinity of ODCase-ZINC70454134 and boosted evidence for its potential efficacy against *A. butzleri*.

It is important to note that the development of new drugs is a long and complex process, and more research is needed to determine the efficacy and safety of potential drug targets. *In silico* ADME/Tox prediction and PBPK simulation were therefore carried out to test various aspects of the possible compound action in body and their safety. Varied profile for simulated compounds in normal vs. pregnant condition was observed and may be due to changes in the physiology of the gastrointestinal tract and/or blood vessels during pregnancy could affect the absorption and distribution of drugs. For example, the reduced gastric acid secretion and increased intestinal motility during pregnancy [58] could lead to altered drug absorption. Previously lower effective exposure and enhanced metabolism of anti HIV-integrases were noted in pregnant women, possibly due to enhanced hormone release [59]. Altered renal or hepatic function during pregnancy could also impact the elimination of drugs from the body, since the glomerular filtration rate increases during pregnancy and may lead to increased clearance of some drugs [60]. In Table 4, it can be seen that the different compounds have different patterns of change between normal and pregnant conditions. Here, TCM compounds had a higher AUC(0-inf) value under pregnant conditions, indicating higher overall exposure and retention for the drug. This may be due to increased absorption and/or reduced metabolism or elimination of the drug during pregnancy. On the other hand, compound ZINC85632684 has a shorter Tmax value under normal conditions, which suggests faster absorption and/or distribution of the drug in nonpregnant individuals. These differences highlight the importance of considering the effects of pregnancy on drug pharmacokinetics, especially for drugs that are used during pregnancy. Most of the studies and clinical trials exclude pregnant women from the study, and little information is available for comparing the impact of various drugs on normal cohort vs. pregnant women. The results of this study and, in general, *in silico* simulation can, therefore, be used to inform further experimental or clinical studies, as well as to make predictions about the likely effects of changing doses, dosing regimens, or physiological conditions. Furthermore, in patients with cirrhosis, renal impairment, and steatosis, there was a decrease in overall bioavailability and absorption rates compared to the general population. This suggests that a smaller fraction of the drug reaches the systemic circulation in individuals with these medical conditions. The first-pass metabolism showed relatively consistent patterns, but AUC values were notably lower in patients with cirrhosis, renal impairment, and steatosis, indicating reduced total exposure to the drugs over time in these populations. Understanding these variations is crucial for tailoring drug regimens to individuals with specific medical conditions. Higher exposure levels in pregnant women may necessitate dosage adjustments to ensure therapeutic efficacy while avoiding potential adverse effects. In patients with cirrhosis, renal impairment, and steatosis, the pronounced decrease in absorption, Cmax, and AUC underscores the importance of individualized treatment approaches to achieve optimal therapeutic outcomes while minimizing the risk of side effects. These pharmacokinetic insights provide valuable guidance for clinicians in optimizing dosing regimens based on the specific physiological conditions and medical histories of patients. However, simulation-based findings may not perfectly replicate the complex dynamics and interactions that occur *in vivo*. Thus, further experimental validation, such as testing in cell lines and animal models, is necessary to confirm the efficacy and safety of drugs and, in this case, ZINC70454134 as a potential therapeutic agent against *A. butzleri*.

Antibiotics are still the first line of defense against severe bacterial infections and are often necessary to treat life-threatening conditions. Moreover, the overuse and misuse of antibiotics have led to the emergence of antibiotic-resistant bacteria, which is a major public health concern [61]. Phytochemicals can offer an alternative or complementary approach to antibiotic therapy, particularly for less severe infections or as a prophylactic measure [62, 63]. Research has shown that phytochemicals can have a range of antibacterial effects, such as inhibiting bacterial growth or disrupting bacterial membranes [64, 65]. Medicinal chemistry and structure-activity relationship studies can be used to further modify the structure of phytochemicals to enhance their antibacterial activity and reduce any potential toxic effects [66, 67]. It is also important to note that the dosage, purity, and quality of phytochemicals can vary, which can affect their effectiveness and safety [68]. Therefore, extensive experimentation and validation are needed before using phytochemicals for the treatment of bacterial infections.

## 5. Conclusion

In conclusion, the pan-genome analysis of the studied *A. butzleri* strains revealed a dynamic genomic landscape, characterized by a significant proportion of dispensable and unique gene clusters. The core genome exhibited conservation, and ODCase enzyme was selected for computational-aided drug design. This target has been studied for other pathogenic organisms as well. Three TCM inhibitors were prioritized and ZINC70454134 depicted strongest binding, compared to the control. All compounds were predicted as safe, but PBPK results were different for normal vs. pregnant cohort, highlighting the importance of considering the effects of pregnancy on drug pharmacokinetics. Dynamics simulations confirmed the stability of the ODCase-ligand complexes, with RMSD values indicating consistent binding over the simulation period. Principal component analysis supported the overall stability and variance of the complex dynamics, further validating the suitability of the selected compounds. The selection of a compound as a potential drug candidate involves a comprehensive evaluation of its pharmacological, toxicological, and physicochemical properties. Key factors include the compound's efficacy in the targeted biological process or disease, safety profile, pharmacokinetics, and bioavailability. ZINC70454134 was selected based on its compliance of these parameters. The *in silico* simulations, as carried out in this study, provide a useful starting point for further investigation and optimization of drug targets. Hence, this multifaceted study provides foundation for development of new natural product inhibitors to control the spread of *A. butzleri* infection and may be replicated for other pathogens. These findings lay groundwork for further experimental validation and drug development efforts against *A. butzleri* as the true effectiveness and safety of the compound can only be determined through rigorous experimentation. Therefore, further testing on cell lines and in model organisms is proposed.

## Figures and Tables

**Table 1:** Strain names, assembly accession, and pan-genome statistics for studied *A. butzleri* strains.

Serial no.	Strain	GenBank accession	CDS	GC content	Core gene number	Dispensable gene number	Unique gene number
3	RM4018	GCA_000014025.1	2259	27.05%	1271	984	4
4	JV22-2011	GCA_000185325.1	2381	26.84%	1271	1038	72
5	7h1h	GCA_000215345.3	2199	27.06%	1271	802	126
6	ED-1	GCA_000284355.1	2158	27.07%	1271	821	66
7	L354	GCA_001010595.1	2242	26.93%	1271	861	110
8	L349	GCA_001010605.1	2284	26.95%	1271	860	153
9	L351	GCA_001010615.1	2246	26.98%	1271	968	7
10	L350	GCA_001010665.1	2246	26.95%	1271	971	4
11	L352	GCA_001010675.1	2249	27.02%	1271	974	4
12	L353	GCA_001010695.1	2202	26.90%	1271	801	130
13	L355	GCA_001010715.1	2219	27.10%	1271	819	129
14	17-1168	GCA_003730245.1	2160	26.92%	1271	785	104
15	6 V	GCA_004283115.1	2196	26.85%	1271	790	135
16	55	GCA_004283125.1	2264	26.79%	1271	818	175
17	JV22-2019	GCA_005886055.1	2263	27.02%	1271	987	5
18	16CS0817-2	GCA_009761295.1	2490	26.97%	1271	944	275
19	16CS0821-2	GCA_009761305.1	2127	27.00%	1271	718	138
20	RMIII	GCA_013363855.1	2370	26.89%	1271	996	103
21	RMI	GCA_013363865.1	2391	26.83%	1271	1043	77
22	NCTC 12481	GCA_900187115.1	2282	27.06%	1271	1003	8
23	MGYG-HGUT-02364	GCA_902386335.1	2244	27.02%	1271	958	15
24	Ab_4511	GCA_902500435.1	2286	27.14%	1271	792	223
25	Ab_4211	GCA_902500445.1	2245	26.98%	1271	822	152
26	Ab_A111	GCA_902500465.1	2090	27.06%	1271	749	70
27	Ab_CR641	GCA_902500475.1	2147	27.00%	1271	763	113
28	Ab_CR502	GCA_902500495.1	2187	26.95%	1271	766	150
29	Ab_2811	GCA_902500545.1	2098	27.08%	1271	758	69
30	Ab_2211	GCA_902500565.1	2298	26.85%	1271	877	150
31	Ab_A103	GCA_902500575.1	2096	27.07%	1271	766	59
32	Ab_CR461	GCA_902500585.1	2281	26.82%	1271	845	165
33	Ab_1711	GCA_902500595.1	2250	27.06%	1271	811	168
34	Ab_CR424	GCA_902500605.1	2210	26.85%	1271	806	133
35	Ab_CR604	GCA_902500635.1	2108	27.06%	1271	799	38
36	Ab_CR1143	GCA_902500645.1	2283	26.88%	1271	825	187
37	Ab_CR891	GCA_902500655.1	2130	26.98%	1271	741	118
38	Ab_CR892	GCA_902500665.1	2267	26.95%	1271	817	179
39	Ab_DQ20dA1	GCA_902500675.1	2297	26.85%	1271	884	142
40	Ab_CR1132	GCA_902500685.1	2517	26.68%	1271	822	424
41	Ab_DQ40A1	GCA_902500765.1	2332	26.86%	1271	873	188
42	Ab_3711	GCA_902500815.1	2160	26.85%	1271	747	142
43	Ab_DQ31A1	GCA_902500965.1	2279	26.77%	1271	880	128
44	Ab_DQ64A1	GCA_902501005.1	2302	26.85%	1271	826	205

**Table 2:** Different interaction scores of compound binding with ODCase.

Compound	Interactions				Δ*G* from PRODIGY (kcal/mol)
	Ligand atom	Receptor residue	Interaction type	Distance (Å)	
Orotidylic acid	O22	ASP8	H-donor	2.74	-5.04
	O1	LYS61	H-acceptor	2.90	
	O16	LYS61	H-acceptor	2.91	
	O22	LYS30	H-acceptor	3.00	
	6-ring	PHE33	pi-H	3.83	
	6-ring	ARG34	pi-H	4.14	

ZINC70454134	—	—	—	—	-5.40

ZINC85632684	N23	Asp8	H-donor	3.16	-5.09
	N25	THR35	H-donor	3.12	
	O32	LYS61	H-acceptor	3.27	
	O30	LYS61	Ionic	3.24	
	O32	LYS61	Ionic	3.48	

ZINC85632721	O33	THR35	H-donor	2.93	-5.25
	O37	LYS61	H-acceptor	3.08	
	O38	LYS61	H-acceptor	2.97	
	O37	LYS61	Ionic	3.08	
	O38	LYS61	Ionic	2.97	

**Table 3:** ADMET parameters of prioritized TCM compounds along with control.

Property	Model name	Control	ZINC70454134	ZINC85632684	ZINC85632721	Unit
Absorption	Water solubility	-3.501	-3.638	-3.112	-3.287	Numeric (log mol/L)
	Caco2 permeability	0.787	0.772	-0.246	0.563	Numeric (log Papp in 10-6 cm/s)
	Intestinal absorption (human)	90.666	57.242	51.569	62.137	Numeric (% absorbed)
	Skin permeability	-2.711	-2.735	-2.735	-2.735	Numeric (log Kp)
	P-Glycoprotein substrate	No	No	Yes	Yes	Categorical (yes/no)
	P-Glycoprotein I inhibitor	No	No	No	No	Categorical (yes/no)
	P-Glycoprotein II inhibitor	No	Yes	Yes	Yes	Categorical (yes/no)

Distribution	VDss (human)	-0.754	-0.459	-0.072	-0.027	Numeric (log L/kg)
	Fraction unbound (human)	0.301	0.072	0.114	0.169	Numeric (Fu)
	BBB permeability	-0.002	-0.986	-1.63	-1.083	Numeric (log BB)
	CNS permeability	-2.814	-2.346	-3.189	-2.963	Numeric (log PS)

Metabolism	CYP2D6 substrate	No	No	No	No	Categorical (yes/no)
	CYP3A4 substrate	No	Yes	Yes	Yes	Categorical (yes/no)
	CYP1A2 inhibitor	No	No	No	No	Categorical (yes/no)
	CYP2C19 inhibitor	No	No	No	No	Categorical (yes/no)
	CYP2C9 inhibitor	No	No	No	No	Categorical (yes/no)
	CYP2D6 inhibitor	No	No	No	No	Categorical (yes/no)
	CYP3A4 inhibitor	No	No	No	No	Categorical (yes/no)

Excretion	Total clearance	1.838	0.494	0.706	0.576	Numeric (log mL/min/kg)
	Renal OCT2 substrate	No	No	No	No	Categorical (yes/no)

Toxicity	AMES toxicity	No	No	No	No	Categorical (yes/no)
	Max. tolerated dose (human)	-0.121	0.437	-0.398	0.172	Numeric (log mg/kg/day)
	hERG I inhibitor	No	No	No	No	Categorical (yes/no)
	hERG II inhibitor	No	No	Yes	Yes	Categorical (yes/no)
	Oral rat acute toxicity (LD50)	2.165	2.857	2.463	2.527	Numeric (mol/kg)
	Oral rat chronic toxicity (LOAEL)	0.202	1.439	2.992	1.973	Numeric (log mg/kg_bw/day)
	Hepatotoxicity	No	No	No	Yes	Categorical (yes/no)
	Skin sensitisation	Yes	No	No	No	Categorical (yes/no)
	T. pyriformis toxicity	0.78	0.285	0.285	0.285	Numeric (log *μ*g/L)
	Minnow toxicity	-1.564	-0.134	-2.73	-0.139	Numeric (log mM)

**Table 4:** PBPK parameters of TCM compounds. AUC(0-inf) represents the total exposure to the compound over time, while AUC(0-t) indicates the AUC from the human exposure to the compound up to the end of the experimental time.

Condition	Compound	Fa (%)	FDp (%)	*F* (%)	Maximum concentration in blood (*μ*g/mL)	Time at which Cmax occurred (h)	AUC(0-inf) (ng-h/mL)	AUC(0-t) (ng-h/mL)
Normal	ZINC70454134	89.19	87.889	87.889	8.13	9.78	5,621,000	67,700
	ZINC85632684	62.20	59.24	59.24	1.13	10	7600.5	7600.5
	ZINC85632721	64.51	62.56	62.56	0.73	10	4792.8	4792.8

Cirrhosis	ZINC70454134	83.57	81.86	81.86	6.67	10	1,410,000	54,490
	ZINC85632684	59.83	56.67	56.67	0.96	9.89	6481.6	6481.6
	ZINC85632721	59.85	57.85	57.85	0.61	9.89	3995.2	3995.2

Renal impairment	ZINC70454134	85.32	83.83	83.83	7.54	9.92	2,148,000	60,460
	ZINC85632684	61.48	58.32	58.32	0.97	9.89	6460.1	6460.1

	ZINC85632721	65.04	63.12	63.12	0.62	10	4125.6	4125.6

Steatosis	ZINC70454134	87.50	86.14	86.14	7.45	9.91	1,169,000	60,680
	ZINC85632684	61.84	58.71	58.713	0.99	9.89	6682	6682
	ZINC85632721	61.15	59.37	59.37	0.64	10	4239.8	4239.8

Pregnant	ZINC70454134	87.48	86.1	86.1	8.98	9.81	8,393,000	72,310
	ZINC85632684	61.11	58.285	58.28	1.14	9.68	7819.4	7819.4
	ZINC85632721	62.115	60.243	60.24	0.75	10	4888.4	4888.4

Fa (%) = fraction of the compound that is absorbed from the gut into the bloodstream; FDp (%) = fraction of the compound that is eliminated from the gut via first-pass metabolism (i.e., the fraction of the absorbed dose that gets metabolized before it can enter the bloodstream); *F* (%) = administered dose that makes it into the bloodstream after accounting for both absorption and first-pass metabolism.

## Data Availability

All the data used or generated in this study is provided as accession number or relevant information as tables in the manuscript.

## Supplementary Materials

Supplementary Materials Supplementary Figure 1: physiology information for pharmacokinetic parameters for (A) normal, (B) cirrhosis, (C) renally impaired, (D) steatosis, and (E) pregnant. Supplementary Figure 2: (A) Nucleic acid variation of core and pan-genome of A. butzleri. (B) COG categorization of pan, core, and accessory/dispensable genome. Supplementary Figure 3: (A) hydrogen bonds, hydrophobic, ionic, and water bridge contacts for the control and ODCase-simulated complex. (B) Interactions that occur more than 30.0% of the simulation time for control-ODCase simulation in the trajectory (0.00 through 100.00 nsec). Supplementary Figure 4: (A) hydrogen bonds, hydrophobic, ionic, and water bridge contacts for the ZINC70454134 and ODCase-simulated complex. (B) Interactions that occur more than 30.0% of the simulation time for ZINC70454134-ODCase simulation in the trajectory (0.00 through 100.00 nsec). (C) Hydrogen bonds, hydrophobic, ionic, and water bridge contacts for the ZINC85632684 and ODCase-simulated complex. (D) Interactions that occur more than 30.0% of the simulation time for ZINC85632684-ODCase simulation in the trajectory (0.00 through 100.00 nsec). (E) Hydrogen bonds, hydrophobic, ionic, and water bridge contacts for the ZINC85632721 and ODCase-simulated complex. (F) Interactions that occur more than 30.0% of the simulation time for ZINC85632721-ODCase simulation in the trajectory (0.00 through 100.00 nsec). Supplementary Figure 5: (A) eigenvalue plot of ODCase-ZINC70454134 complex. (B) Eigenvalue plot of ODCase-ZINC85632684 complex. (C) Eigenvalue plot of ODCase-ZINC85632721 complex. Supplementary Figure 6: (A) RMSD plot of ODCase-control complex for 100 ns. Ligand trajectory is shown in red and receptor in green. R1 depicts replica 1. (B) RMSD plot of ODCase-control complex for 100 ns. R2 depicts replica 2. (C) RMSD plot of ODCase-ZINC70454134 complex for 100 ns. R1 depicts replica 1. (D) RMSD plot of ODCase-ZINC70454134 complex for 100 ns. R2 depicts replica 2. (E) RMSD plot of ODCase-ZINC85632684 complex for 100 ns. R1 depicts replica 1. (F) RMSD plot of ODCase-ZINC85632684 complex for 100 ns. R2 depicts replica 2. (G) RMSD plot of ODCase-ZINC85632721 complex for 100 ns. R1 depicts replica 1. (H) RMSD plot of ODCase-ZINC85632721 complex for 100 ns. R2 depicts replica 2. Supplementary Table 1: KEGG pathways of drug targets mined from the core genome of A. butzleri. Empty cells indicate no information.
