# Supplementary material for: Exploring Lead-Like Molecules of Traditional Chinese Medicine for Treatment Quest against Aliarcobacter butzleri: In Silico Toxicity Assessment, Dynamics Simulation, and Pharmacokinetic Profiling
Source: Biomed Res Int. 2024 Feb 22;2024:9377016. doi: 10.1155/2024/9377016 (PMC11401669; doi:10.1155/2024/9377016)
Supplement: Supplementary Materials — Supplementary Figure 1: physiology information for pharmacokinetic parameters for (A) normal, (B) cirrhosis, (C) renally impaired, (D) steatosis, and (E) pregnant. Supplementary Figure 2: (A) Nucleic acid variation of core and pan-genome of A. butzleri. (B) COG categorization of pan, core, and accessory/dispensable genome. Supplementary Figure 3: (A) hydrogen bonds, hydrophobic, ionic, and water bridge contacts for the control and ODCase-simulated complex. (B) Interactions that occur more than 30.0% of the simulation time for control-ODCase simulation in the trajectory (0.00 through 100.00 nsec). Supplementary Figure 4: (A) hydrogen bonds, hydrophobic, ionic, and water bridge contacts for the ZINC70454134 and ODCase-simulated complex. (B) Interactions that occur more than 30.0% of the simulation time for ZINC70454134-ODCase simulation in the trajectory (0.00 through 100.00 nsec). (C) Hydrogen bonds, hydrophobic, ionic, and water bridge contacts for the ZINC85632684 and ODCase-simulated complex. (D) Interactions that occur more than 30.0% of the simulation time for ZINC85632684-ODCase simulation in the trajectory (0.00 through 100.00 nsec). (E) Hydrogen bonds, hydrophobic, ionic, and water bridge contacts for the ZINC85632721 and ODCase-simulated complex. (F) Interactions that occur more than 30.0% of the simulation time for ZINC85632721-ODCase simulation in the trajectory (0.00 through 100.00 nsec). Supplementary Figure 5: (A) eigenvalue plot of ODCase-ZINC70454134 complex. (B) Eigenvalue plot of ODCase-ZINC85632684 complex. (C) Eigenvalue plot of ODCase-ZINC85632721 complex. Supplementary Figure 6: (A) RMSD plot of ODCase-control complex for 100 ns. Ligand trajectory is shown in red and receptor in green. R1 depicts replica 1. (B) RMSD plot of ODCase-control complex for 100 ns. R2 depicts replica 2. (C) RMSD plot of ODCase-ZINC70454134 complex for 100 ns. R1 depicts replica 1. (D) RMSD plot of ODCase-ZINC70454134 complex for 100 ns. R2 depicts replica 2. (E) RMSD p [file 9377016.f1.docx]

**Supplementary Table 1.** KEGG pathways of drug targets mined from the core genome of *A. butzleri.* Empty cells indicate no information.

| Accession ID | No. of amino acids | KO number | Description | Score | Pathway |
| --- | --- | --- | --- | --- | --- |
| WP_004509153.1 | 184 | K03186 | ubiX, bsdB, PAD1; flavin prenyltransferase [EC:2.5.1.129] | 234 | map00130 Ubiquinone and other terpenoid-quinone biosynthesis map00627 Aminobenzoate degradation map00740 Riboflavin metabolism map00900 Terpenoid backbone biosynthesis map00940 Phenylpropanoid biosynthesis map01100 Metabolic pathways map01110 Biosynthesis of secondary metabolites map01120 Microbial metabolism in diverse environments map01220 Degradation of aromatic compounds map01240 Biosynthesis of cofactors |
| WP_004509380.1 | 280 | K00575 | cheR; chemotaxis protein methyltransferase CheR [EC:2.1.1.80] | 222 | map02020 Two-component system map02030 Bacterial chemotaxis |
| WP_004510359.1 | 266 | K25996 | frdC, fdrC; succinate dehydrogenase subunit C | 239 | map00020 Citrate cycle (TCA cycle) map00190 Oxidative phosphorylation map00620 Pyruvate metabolism map00650 Butanoate metabolism map00720 Carbon fixation pathways in prokaryotes map01100 Metabolic pathways map01110 Biosynthesis of secondary metabolites map01120 Microbial metabolism in diverse environments |
| WP_004510492.1 | 127 | K01579 | panD; aspartate 1-decarboxylase [EC:4.1.1.11] | 147 | map00410 beta-Alanine metabolism map00770 Pantothenate and CoA biosynthesis map01100 Metabolic pathways map01110 Biosynthesis of secondary metabolites map01240 Biosynthesis of cofactors |
| WP_004510707.1 | 261 | K03474 | pdxJ; pyridoxine 5-phosphate synthase [EC:2.6.99.2] | 269 | map00750 Vitamin B6 metabolism map01100 Metabolic pathways map01240 Biosynthesis of cofactors |
| WP_004511102.1 | 260 | K00677 | lpxA; UDP-N-acetylglucosamine acyltransferase [EC:2.3.1.129] | 323 | map00540 Lipopolysaccharide biosynthesis map01100 Metabolic pathways map01503 Cationic antimicrobial peptide (CAMP) resistance |
| WP_004511216.1 | 235 | K15256 | cmoA; tRNA (cmo5U34)-methyltransferase [EC:2.1.1.-] | 250 |  |
| WP_012012516.1 | 222 | K21929 | udg; uracil-DNA glycosylase [EC:3.2.2.27] | 154 | map03410 Base excision repair |
| WP_014467954.1 | 1035 | K07787 | cusA, silA; copper/silver efflux system protein | 1000 | map02020 Two-component system |
| WP_014467955.1 | 187 |  |  | 121 |  |
| WP_014468021.1 | 672 | K03406 | mcp; methyl-accepting chemotaxis protein | 39 | map02020 Two-component system map02030 Bacterial chemotaxis |
| WP_014468039.1 | 341 | K01465 | URA4, pyrC; dihydroorotase [EC:3.5.2.3] | 345 | ap00240 Pyrimidine metabolism map01100 Metabolic pathways map01240 Biosynthesis of cofactors |
| WP_014468053.1 | 272 | K01918 | panC; pantoate--beta-alanine ligase [EC:6.3.2.1] | 278 | map00410 beta-Alanine metabolism map00770 Pantothenate and CoA biosynthesis map01100 Metabolic pathways map01110 Biosynthesis of secondary metabolites map01240 Biosynthesis of cofactors |
| WP_014468130.1 | 701 | K02014 | TC.FEV.OM; iron complex outermembrane recepter protein | 382 |  |
| WP_014468141.1 | 496 | K03385 | nrfA; nitrite reductase (cytochrome c-552) [EC:1.7.2.2] | 441 | map00910 Nitrogen metabolism map01100 Metabolic pathways map01120 Microbial metabolism in diverse environments |
| WP_014468144.1 | 538 | K20964 | cdpA; c-di-GMP phosphodiesterase [EC:3.1.4.52] | 62 | map05111 Biofilm formation - Vibrio cholerae |
| WP_014468224.1 | 1008 | K03296 | TC.HAE1; hydrophobic/amphiphilic exporter-1 (mainly G- bacteria), HAE1 family | 683 |  |
| WP_014468276.1 | 633 | K03406 | mcp; methyl-accepting chemotaxis protein | 317 | map02020 Two-component system map02030 Bacterial chemotaxis |
| WP_014468339.1 | 627 | K03406 | mcp; methyl-accepting chemotaxis protein | 101 | map02020 Two-component system map02030 Bacterial chemotaxis |
| WP_014468343.1 | 463 | K03406 | mcp; methyl-accepting chemotaxis protein | 19 | map02020 Two-component system map02030 Bacterial chemotaxis |
| WP_014468348.1 | 769 | K03406 | mcp; methyl-accepting chemotaxis protein | 298 | map02020 Two-component system map02030 Bacterial chemotaxis |
| WP_014468359.1 | 311 | K00097 | pdxA; 4-hydroxythreonine-4-phosphate dehydrogenase [EC:1.1.1.262] | 396 | map00750 Vitamin B6 metabolism map01100 Metabolic pathways map01240 Biosynthesis of cofactors |
| WP_014468390.1 | 373 |  |  |  |  |
| WP_014468430.1 | 730 | K03406 | mcp; methyl-accepting chemotaxis protein | 355 | map02020 Two-component system map02030 Bacterial chemotaxis |
| WP_014468432.1 | 239 |  |  | 175 |  |
| WP_014468447.1 | 753 | K03406 | mcp; methyl-accepting chemotaxis protein | 249 | map02020 Two-component system map02030 Bacterial chemotaxis |
| WP_014468450.1 | 798 | K03406 | mcp; methyl-accepting chemotaxis protein | 308 | map02020 Two-component system map02030 Bacterial chemotaxis |
| WP_014468456.1 | 1042 | K18138 | acrB, mexB, adeJ, smeE, mtrD, cmeB; multidrug efflux pump | 944 |  |
| WP_014468476.1 | 466 | K03406 | mcp; methyl-accepting chemotaxis protein | 219 | map02020 Two-component system map02030 Bacterial chemotaxis |
| WP_014468523.1 | 502 | K03406 | mcp; methyl-accepting chemotaxis protein | 60 | map02020 Two-component system map02030 Bacterial chemotaxis |
| WP_014468616.1 | 785 | K02014 | TC.FEV.OM; iron complex outermembrane recepter protein | 283 |  |
| WP_014468680.1 | 211 |  |  | 259 |  |
| WP_014468700.1 | 222 | K00684 | aat; leucyl/phenylalanyl-tRNA---protein transferase [EC:2.3.2.6] | 190 |  |
| WP_014468738.1 | 827 | K07812 | torZ; trimethylamine-N-oxide reductase (cytochrome c) [EC:1.7.2.3] | 638 | map00680 Methane metabolism map01100 Metabolic pathways map01120 Microbial metabolism in diverse environments |
| WP_014468748.1 | 315 | K02536 | lpxD; UDP-3-O-[3-hydroxymyristoyl] glucosamine N-acyltransferase [EC:2.3.1.191] | 298 | map00540 Lipopolysaccharide biosynthesis map01100 Metabolic pathways |
| WP_014468812.1 | 243 | K00979 | kdsB; 3-deoxy-manno-octulosonate cytidylyltransferase (CMP-KDO synthetase) [EC:2.7.7.38] | 239 | map00540 Lipopolysaccharide biosynthesis map01100 Metabolic pathways map01250 Biosynthesis of nucleotide sugars |
| WP_014468846.1 | 730 | K00031 | IDH1, IDH2, icd; isocitrate dehydrogenase [EC:1.1.1.42] | 806 | map00020 Citrate cycle (TCA cycle) map00480 Glutathione metabolism map00720 Carbon fixation pathways in prokaryotes map01100 Metabolic pathways map01110 Biosynthesis of secondary metabolites map01120 Microbial metabolism in diverse environments map01200 Carbon metabolism map01210 2-Oxocarboxylic acid metabolism map01230 Biosynthesis of amino acids map04146 Peroxisome map05230 Central carbon metabolism in cancer |
| WP_014468917.1 | 657 | K03406 | mcp; methyl-accepting chemotaxis protein | 255 | map02020 Two-component system map02030 Bacterial chemotaxis |
| WP_014468956.1 | 782 | K02014 | TC.FEV.OM; iron complex outermembrane recepter protein | 244 |  |
| WP_014468957.1 | 479 |  |  | 279 |  |
| WP_014469026.1 | 735 | K03782 | katG; catalase-peroxidase [EC:1.11.1.21] | 825 | map00360 Phenylalanine metabolism map00380 Tryptophan metabolism map00940 Phenylpropanoid biosynthesis map00983 Drug metabolism - other enzymes map01100 Metabolic pathways map01110 Biosynthesis of secondary metabolites |
| WP_014469032.1 | 851 |  |  | 561 |  |
| WP_014469110.1 | 595 | K03406 | mcp; methyl-accepting chemotaxis protein | 113 | map02020 Two-component system map02030 Bacterial chemotaxis |
| WP_014469127.1 | 707 | K03406 | mcp; methyl-accepting chemotaxis protein | 122 | map02020 Two-component system map02030 Bacterial chemotaxis |
| WP_014469159.1 | 269 | K00606 | panB; 3-methyl-2-oxobutanoate hydroxymethyltransferase [EC:2.1.2.11] | 286 | map00770 Pantothenate and CoA biosynthesis map01100 Metabolic pathways map01110 Biosynthesis of secondary metabolites map01240 Biosynthesis of cofactors |
| WP_014469175.1 | 189 | K03271 | gmhA, lpcA; D-sedoheptulose 7-phosphate isomerase [EC:5.3.1.28] | 210 | map00540 Lipopolysaccharide biosynthesis map01100 Metabolic pathways map01250 Biosynthesis of nucleotide sugars |
| WP_014469176.1 | 632 |  |  | 363 |  |
| WP_014469264.1 | 970 |  |  | 65 |  |
| WP_014469323.1 | 528 | K00392 | sir; sulfite reductase (ferredoxin) [EC:1.8.7.1] | 372 | map00920 Sulfur metabolism map01100 Metabolic pathways map01120 Microbial metabolism in diverse environments |
| WP_014469331.1 | 755 | K00549 | metE; 5-methyltetrahydropteroyltriglutamate--homocysteine methyltransferase [EC:2.1.1.14] | 733 | map00270 Cysteine and methionine metabolism map00450 Selenocompound metabolism map01100 Metabolic pathways map01110 Biosynthesis of secondary metabolites map01230 Biosynthesis of amino acids |
| WP_014469339.1 | 301 | K02535 | lpxC; UDP-3-O-[3-hydroxymyristoyl] N-acetylglucosamine deacetylase [EC:3.5.1.108] | 307 | map00540 Lipopolysaccharide biosynthesis map01100 Metabolic pathways |
| WP_014469365.1 | 488 | K00404 | ccoN; cytochrome c oxidase cbb3-type subunit I [EC:7.1.1.9] | 631 | map00190 Oxidative phosphorylation map01100 Metabolic pathways map02020 Two-component system |
| WP_014469418.1 | 581 |  |  | 21 |  |
| WP_014469422.1 | 776 | K02014 | TC.FEV.OM; iron complex outermembrane recepter protein | 330 |  |
| WP_014469474.1 | 203 | K00793 | ribE, RIB5; riboflavin synthase [EC:2.5.1.9] | 224 | map00740 Riboflavin metabolism map01100 Metabolic pathways map01110 Biosynthesis of secondary metabolites map01240 Biosynthesis of cofactors |
| WP_014469485.1 | 396 | K07552 | bcr, tcaB; MFS transporter, DHA1 family, multidrug resistance protein | 160 |  |
| WP_080584782.1 | 933 | K00123 | fdoG, fdhF, fdwA; formate dehydrogenase major subunit [EC:1.17.1.9] | 824 | map00630 Glyoxylate and dicarboxylate metabolism map00680 Methane metabolism map01100 Metabolic pathways map01120 Microbial metabolism in diverse environments map01200 Carbon metabolism |
| WP_198407793.1 | 228 | K01591 | pyrF; orotidine-5'-phosphate decarboxylase [EC:4.1.1.23] | 325 | map00240 Pyrimidine metabolism map01100 Metabolic pathways map01240 Biosynthesis of cofactors |
| WP_228126984.1 | 372 | K07552 | bcr, tcaB; MFS transporter, DHA1 family, multidrug resistance protein | 201 |  |


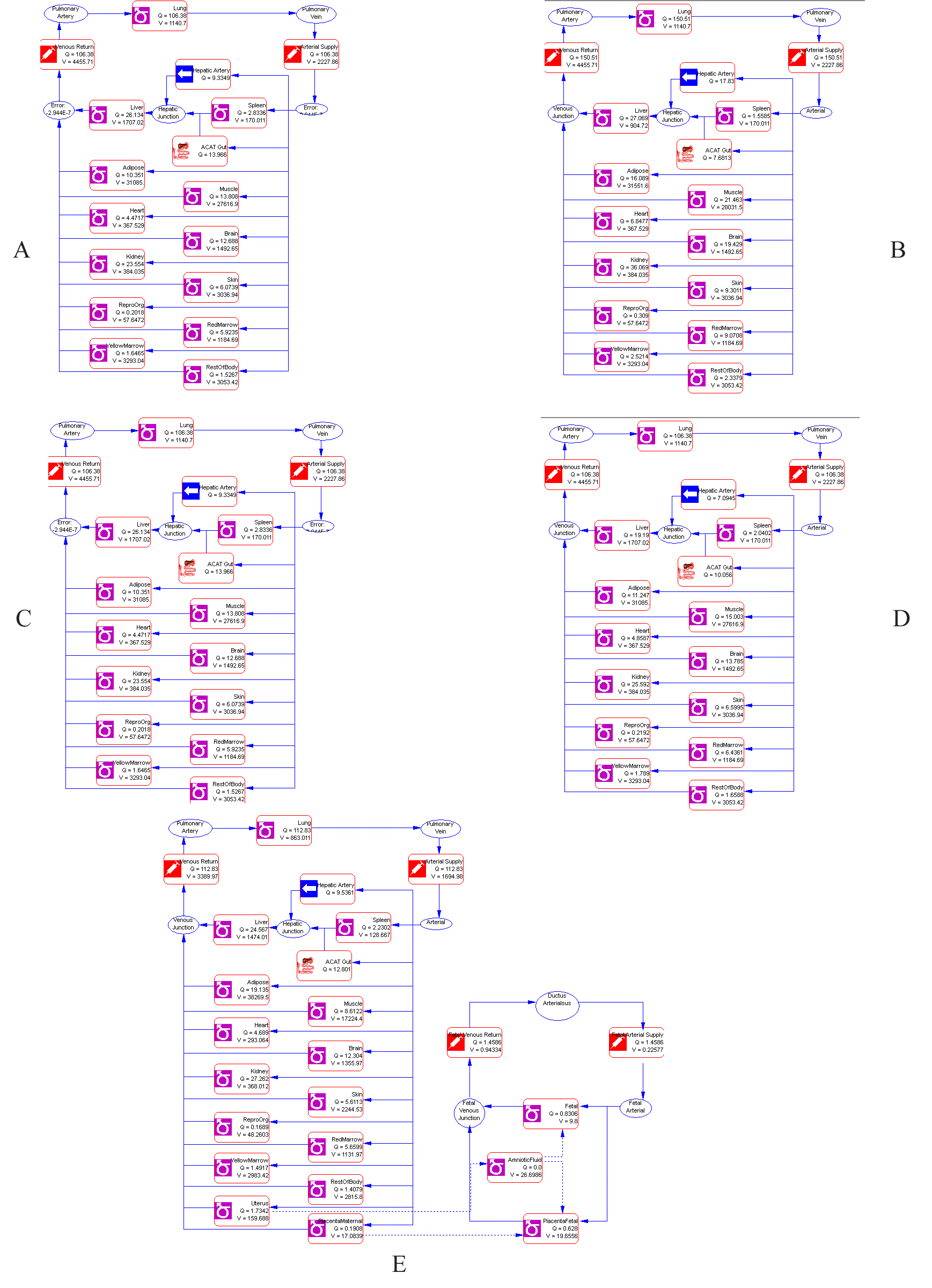
**Supplementary Fig. 1**. Physiology information for pharmacokinetic parameters for (A) Normal (B) Cirrhosis (C) Renally impaired (D) Steatosis (E)Pregnant.


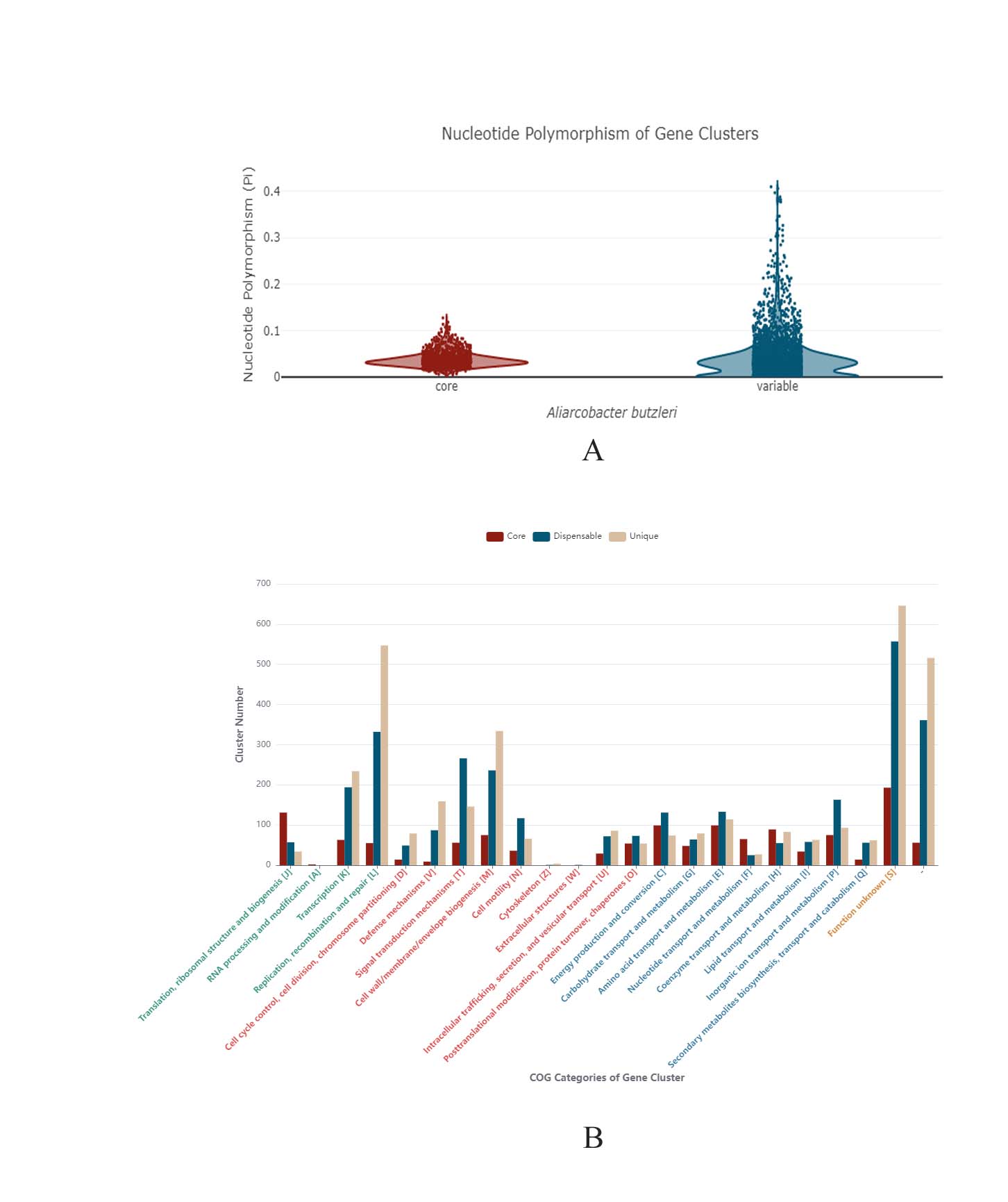


**Supplementary Fig. 2.** (A). Nucleic acid variation of core and pan-genome of *A. butzleri.* (B). COG categorization of pan, core and accessory/dispensable genome.

**
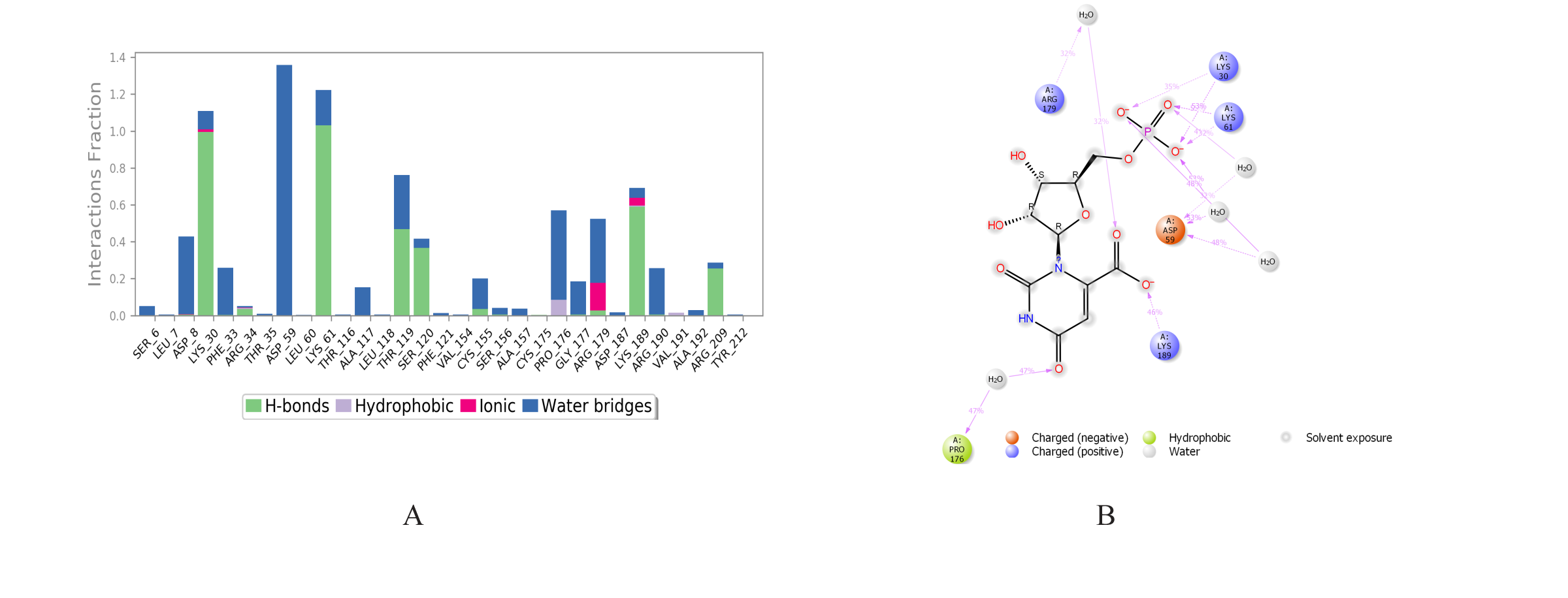
**

**Supplementary Fig. 3** (A) Hydrogen bonds, hydrophobic, ionic and water bridges contacts for the control and ODCase simulated complex (B) Interactions that occur more than 30.0% of the simulation time for control-ODCase simulation in the trajectory ( 0.00 through 100.00 nsec).

**
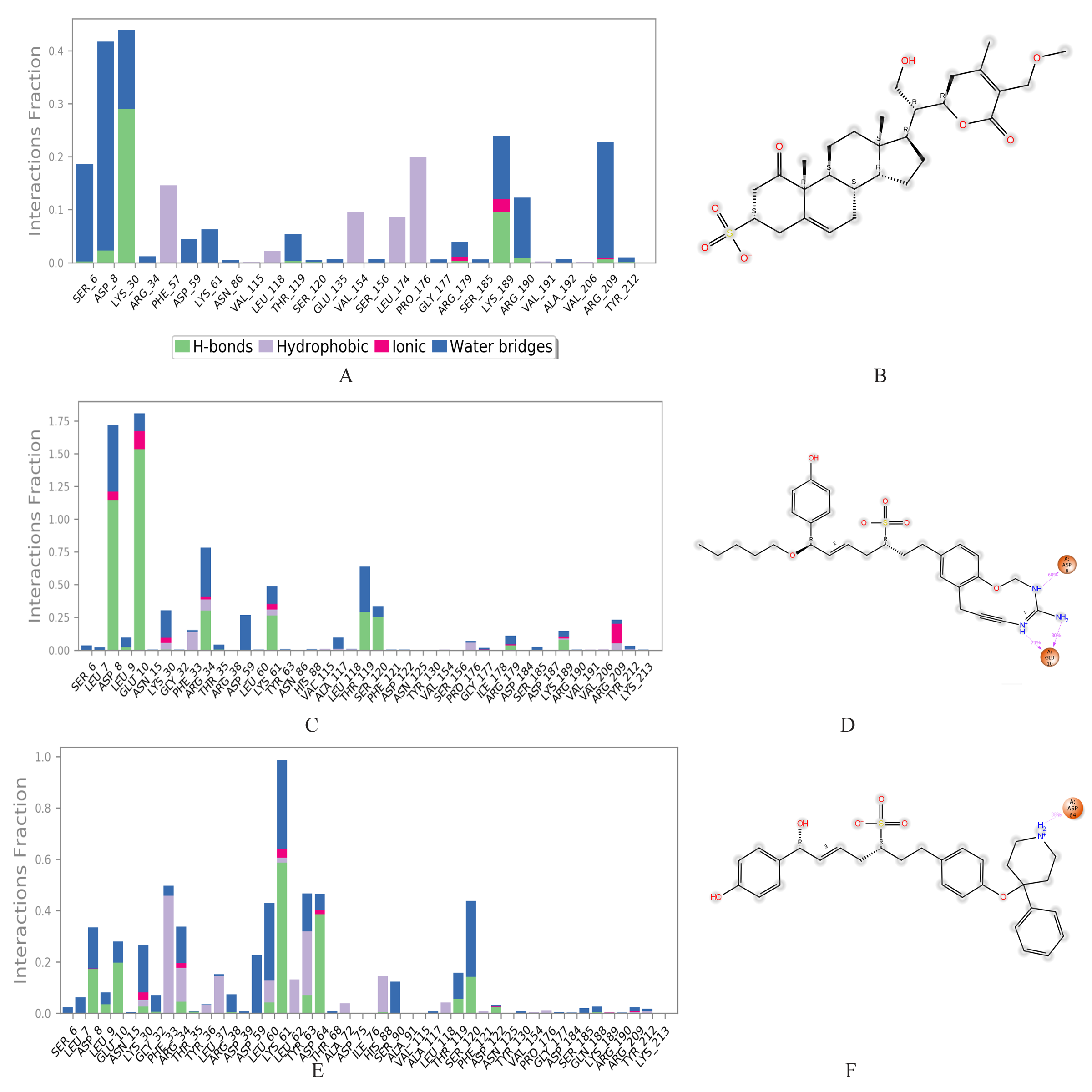
**

**Supplementary Fig. 4** (A) Hydrogen bonds, hydrophobic, ionic and water bridges contacts for the ZINC70454134 and ODCase simulated complex (B) Interactions that occur more than 30.0% of the simulation time for ZINC70454134-ODCase simulation in the trajectory ( 0.00 through 100.00 nsec). (C) Hydrogen bonds, hydrophobic, ionic and water bridges contacts for the ZINC85632684 and ODCase simulated complex (D) Interactions that occur more than 30.0% of the simulation time for ZINC85632684-ODCase simulation in the trajectory (0.00 through 100.00 nsec). (E) Hydrogen bonds, hydrophobic, ionic and water bridges contacts for the ZINC85632721 and ODCase simulated complex (F) ) Interactions that occur more than 30.0% of the simulation time for ZINC85632721-ODCase simulation in the trajectory (0.00 through 100.00 nsec).


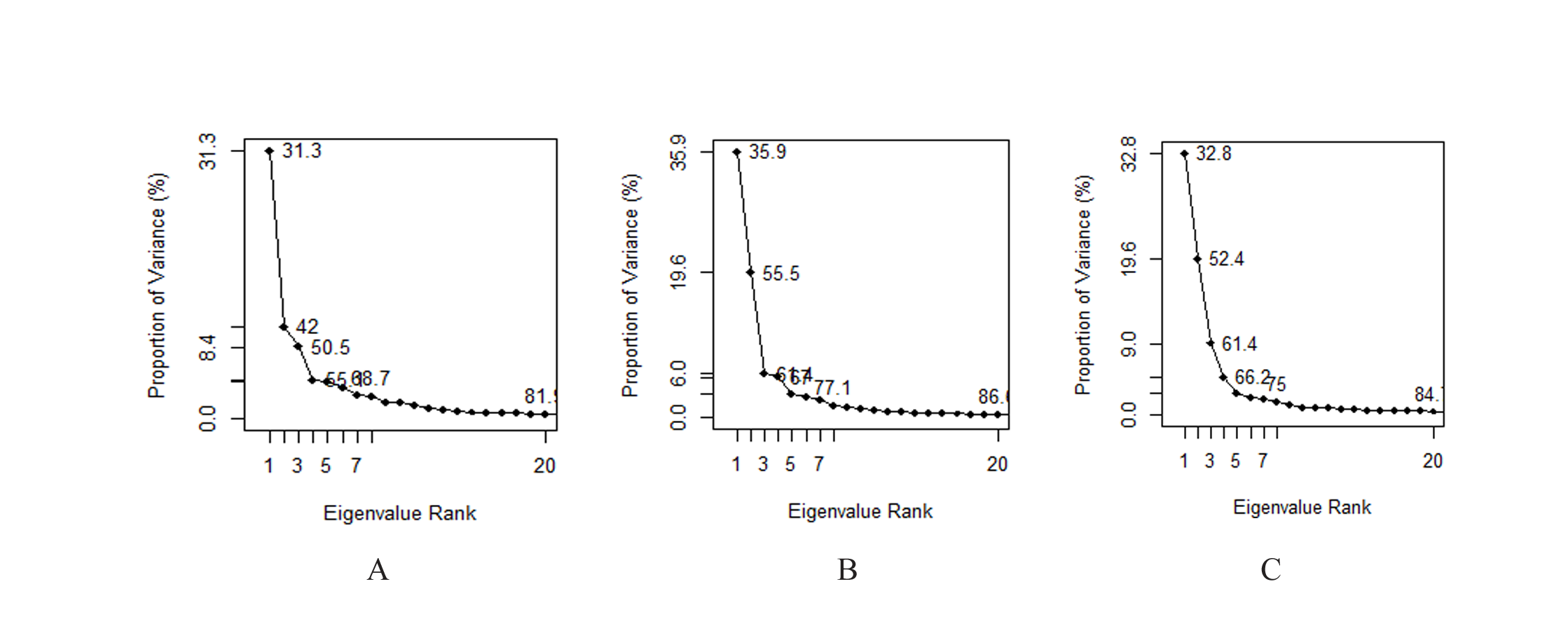


**Supplementary Fig. 5.** (A) Eigenvalue plot of ODCase-ZINC70454134 complex (B) Eigenvalue plot of ODCase- ZINC85632684 complex (C) Eigenvalue plot of ODCase- ZINC85632721 complex.


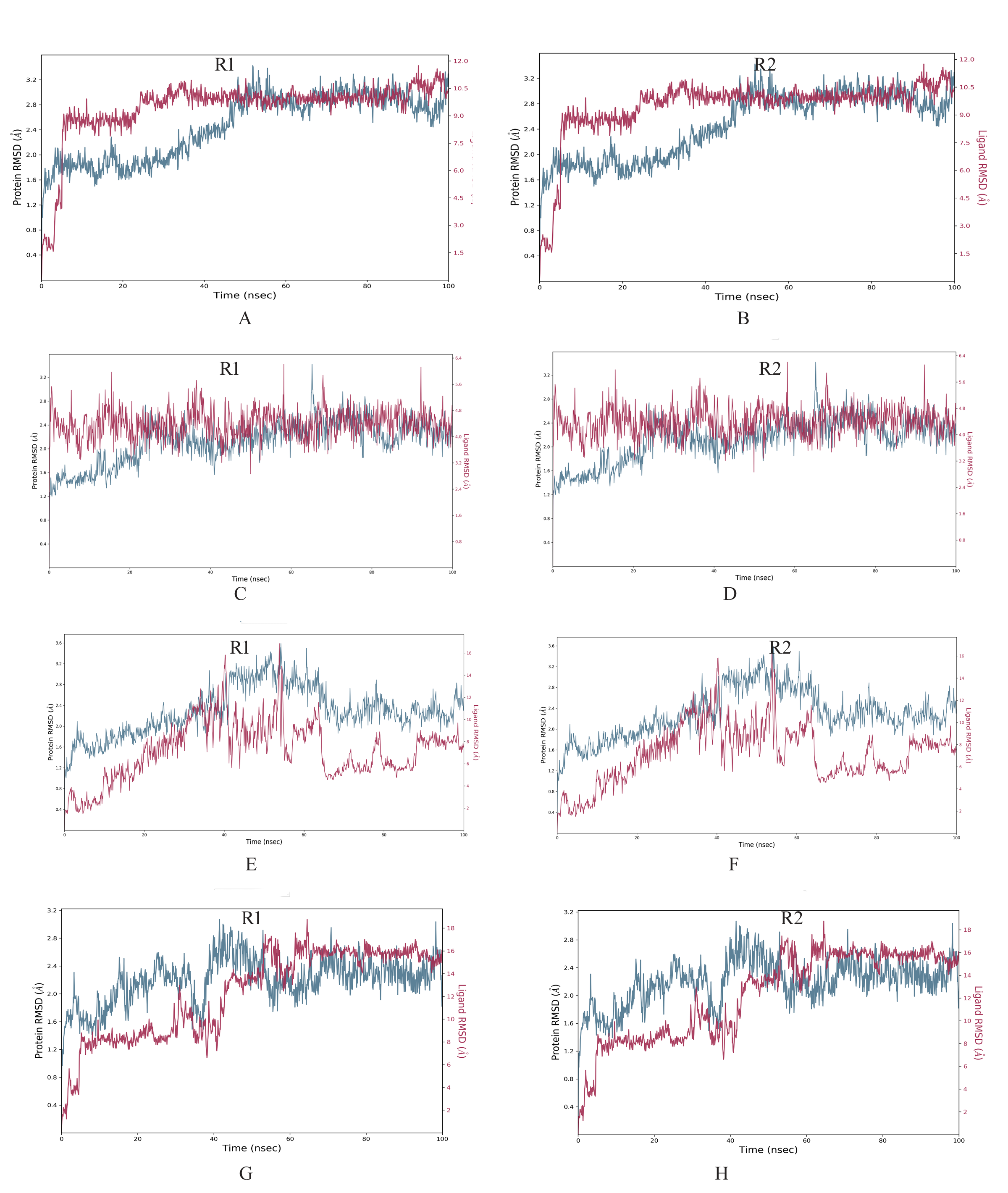


**Supplementary Fig. 6.** (A) RMSD plot of ODCase-control complex for 100 ns. Ligand trajectory is shown by red and receptor by green. R1 depicts replica 1. (B) RMSD plot of ODCase-control complex for 100 ns. R2 depicts replica 2. (C) RMSD plot of ODCase- ZINC70454134 complex for 100 ns. R1 depicts replica 1. (D) RMSD plot of ODCase-ZINC70454134 complex for 100 ns. R2 depicts replica 2. (E) RMSD plot of ODCase - ZINC85632684 complex for 100 ns. R1 depicts replica 1. (F) RMSD plot of ODCase-ZINC85632684 complex for 100 ns. R2 depicts replica 2. (G) RMSD plot of ODCase-ZINC85632721 complex for 100 ns. R1 depicts replica 1. (H) RMSD plot of ODCase-ZINC85632721 complex for 100 ns. R2 depicts replica 2.
